# Reliability of clinical judgment for evaluation of informed consent in mental health settings and the validation of the Evaluation of Informed Consent to Treatment (EICT) scale

**DOI:** 10.3389/fpsyg.2024.1309909

**Published:** 2024-03-19

**Authors:** Nicola Di Fazio, Donato Morena, Federica Piras, Fabrizio Piras, Nerisa Banaj, Giuseppe Delogu, Felice Damato, Paola Frati, Vittorio Fineschi, Stefano Ferracuti, Gabriele Sani, Claudia Dacquino

**Affiliations:** ^1^Department of Anatomical, Histological, Forensic and Orthopaedic Sciences, Sapienza University of Rome, Rome, Italy; ^2^Department of Clinical Neuroscience and Neurorehabilitation, IRCCS Santa Lucia Foundation, Rome, Italy; ^3^Department of Human Neuroscience, Sapienza University of Rome, Rome, Italy; ^4^Department of Psychiatry, Department of Neuroscience, Head, Neck and Thorax, Fondazione Policlinico Universitario Agostino Gemelli IRCCS, Rome, Italy

**Keywords:** informed consent, clinical reliability, competence, mental capacity, schizophrenia, assessment tools, informed consent to treatment scale, EICT scale

## Abstract

**Introduction:**

The competence assessment to give informed consent in the legal and healthcare settings is often performed merely through clinical judgment. Given the acknowledged limited reliability of clinician-based evaluation in the mental health sector, particularly for the assessment of competence to consent, our objective was to ascertain the dependability of clinical judgment when evaluating the ability of schizophrenia patients to make choices about their health.

**Methods:**

The potential convergence between clinical evaluation and scores from a new standardized assessment (the “Evaluation of Informed Consent to Treatment” - “EICT” scale) was therefore tested. The scale assesses four dimensions of competence, specifically how patients normally understand information relating to care (Understanding); how they evaluate the choice of treatment in terms of risk/benefit ratio (Evaluating); how they reason coherently in the decision-making process (Reasoning); and, finally, their ability to make a choice between treatment alternatives (Expressing a choice). Thirty-four outpatients with schizophrenia were evaluated for their competence to consent by five referring clinicians with different backgrounds (psychiatrist, forensic psychiatrist, geriatrician, anesthetist, and medico-legal doctor). Inter-raters variability was tested through correlation analyses between the scores obtained by the clinicians on a modified version of the Global Assessment of Functioning scale (GAF) designed specifically to subjectively assess functioning in each of the four competence dimensions. Two validated competence scales (Mac-CAT-T, SICIATRI-R), and a neuropsychological battery were also administered along with scales for evaluating neuropsychiatric symptoms severity and side effects of medication.

**Results:**

Clinical judgments of the individual specialists showed great inter-rater variability. Likewise, only weak/non-significant correlations were found between the EICT subscales and the respective clinicians-rated GAF scales. Conversely, solid correlations were found between the EICT and MacCAT-T subscales. As expected, healthy controls performed better in the ability to give informed consent to treatment, as measured by the three scales (i.e., EICT, MacCAT-T, and SICIATRI-R), and neuropsychological test performance. In the comparisons between patients who, according to the administered EICT, were able or not able to give informed consent to treatment, significant differences emerged for the Phonemic verbal fluency task (*p* = 0.038), Verbal judgments (*p* = 0.048), MacCAT-T subscales, and SICIATRI-R total score. Moreover, EICT exhibited excellent internal consistency (Cronbach’s alphas ranging from 0.96 to 0.98 for the four subscales) while the Item Analysis, by measuring the correlation between each item of the EICT and the total score, was excellent for all items of all subscales (alphas ranging from 0.86 to 0.98).

**Discussion:**

In conclusion, our findings highlighted that the assessment of competence exclusively through clinical judgment is not fully reliable and needs the support of standardized tools. The EICT scale could therefore be useful in assessing general competence to consent both in healthcare and legal contexts, where it might be necessary to evaluate the effective competence of patients with psychiatric disorders. Finally, this scale could serve as a valuable tool for decisions regarding whether and to what extent a patient needs support.

## 1 Introduction

Informed consent constitutes a crucial aspect of medical practice, signifying the patient’s voluntary and uncoerced acceptance of medical treatment.

In Italy, based on Articles 2, 13, and 32 of the Italian Constitution and Articles 1, 2, and 3 of the Charter of Fundamental Rights of the European Union, Law No. 219 of 2017 (“Rules on Informed Consent and Advance Healthcare Directives”) stipulates that no healthcare treatment can be initiated or continued without the free and informed consent of the individual concerned, except in cases expressly provided by law. Article 1 of this Law, titled “Informed Consent”, establishes the right of every person to be fully informed about their health conditions. Individuals have the right to receive up-to-date and comprehensive information regarding the diagnosis, prognosis, benefits, and risks associated with diagnostic assessments and recommended healthcare treatments. This includes information about possible alternatives and the consequences of refusing healthcare treatment or diagnostic assessments, as well as the option to renounce them. As per various rulings of the Italian Court of Cassation, consent must always be complete and effective, explicitly obtained from the patient. It should be current and informed, indicating that the patient is fully aware of the implications.

Consent must be based on detailed information provided by the physician, and in cases of alleged patient non-compliance, the burden of proof lies with the physician to demonstrate fulfillment of this obligation.

The features of informed consent are also widely stipulated at the international level (Palmer et al., 2005).

The right of persons with severe mental disorders to have full rights and responsibilities, just like all Italian citizens, was established almost fifty years ago by the pioneering Law No. 180 of 1978, commonly known as the “Basaglia Law”. Among various innovations, Law No. 180 established that coercion for individuals with mental disorders is permissible only if (i) there are psychiatric alterations, (ii) requiring urgent therapeutic interventions, and (iii) provided these interventions are not accepted by the patients. Moreover, with this Law the connection between a psychiatric diagnosis and outcomes such as dangerousness and incapacity has been eliminated.

Since the 70s (Roth et al., 1977), in the psychiatric field numerous studies have been dedicated to investigating the neuropsychological foundations of consent. Nevertheless, the issue remains regarding how to assess the validity of consent/refusal given by patients, that is, the identification of valid tools for the accurate assessment of competence (Mandarelli et al., 2012).

Cognitive impairments, perception, and mood disturbances, and thought and behavior abnormalities, as features of various mental disorders, are risk factors for alterations in the decision-making processes underlying the selection and choice of treatment options. Some symptomatic conditions, such as those shown in the acute phases of affective or psychotic disorders, can hinder the ability to consent, along with cognitive impairment affecting the ability to concentrate, understand, assimilate information, and have constancy in decisions (Gupta and Kharawala, 2012). However, mental disorders also constitute a useful model for studying the different processes underlying consent or refusal to treatment (Roberts, 2002). The stereotyped consideration of people with mental disorders as characterized by an intrinsic limitation of their own decision-making capacity has been deeply challenged by scientific literature (Appelbaum, 2006; Palmer et al., 2007; Owen et al., 2008). Even for psychotic disorders, where reality testing could be more problematic, the assumption that the diagnostic condition necessarily entails a state of incapacity at the juridical level has been questioned (Carpenter et al., 2000). However, the decision-making difficulties of patients with severe psychiatric disorders, such as schizophrenia, should be carefully considered. Schizophrenia is deeply burdened by impairments in different cognitive abilities, some of which are strategically involved in the competence to consent (Carpenter et al., 2000; Moser et al., 2002; Kovnick et al., 2003; Palmer et al., 2004; Raymont et al., 2004; Palmer et al., 2007; Gupta and Kharawala, 2012).

According to several authors, the impairment of decision-making in schizophrenia could be subtended by different cognitive deficits, such as those observables in the disorder in short- and long-term memory, attention, and abstract reasoning (Carpenter et al., 2000; Moser et al., 2002; Kovnick et al., 2003). There is, however, little experimental evidence for the hypothesis that particular neurocognitive domains, such as attention, working memory, learning, episodic recall, and more generally executive functions, may be differentially related to specific elements of the decision-making capacity involved in competence to consent.

Studies that used comprehensive batteries of cognitive assessment (Carpenter et al., 2000; Moser et al., 2002; Stroup et al., 2005; Palmer and Jeste, 2006) demonstrated the existence of a relationship between global cognitive functioning and the ability to give consent, but no clear relationships between specific cognitive domains and the dimensions involved in competence emerged.

Generally, an evaluation of competence should be performed accurately according to the standards identified by the scientific literature, investigating the four fundamental dimensions that underlie the decisional capacity to consent to the therapeutic process (Appelbaum and Grisso, 1988; Grisso and Appelbaum, 1998; Carpenter et al., 2000; Appelbaum, 2006), namely: (i) the ability to understand the relevant elements of one’s medical situation and all the information useful for therapeutic choices (Understanding); (ii) the ability to use this information for assessing the implications of the pathological diagnosis (e.g., probable consequences) (Appreciating/Evaluating); (iii) the ability to reason on available information by organizing it in a logical-rational sequence (evaluation of pros and cons), which implies the ability to appraise possible therapeutic alternatives (Reasoning); and (iv) the capacity to express a choice, or to identify someone who can help in making the most appropriate decision (Expressing a choice).

Despite its complexity, the evaluation of informed consent in healthcare settings is often performed without standardized tools. Literature data suggest the poor reliability of clinical judgments in the field of forensic psychiatry, specifically for the assessment of competence to consent, as it is influenced by several biases (Miller, 2001; Miller et al., 2001; Gowensmith et al., 2013).

The risk of mistakes in the evaluation lies in the potential adoption of a paternalistic approach by physicians toward patients, exerting excessive influence on their choices. Conversely, patients’ challenges in evaluating alternatives or articulating preferences may be minimized, potentially leaving them unsupported when confronted with complex decisions.

A useful instrument for a formal investigation of competence to consent to treatment is the MacArthur Competence Assessment Tool for Treatment (MacCAT-T) (Grisso et al., 1997) which allows the assessment of the four cognitive areas listed above. However, this tool does have certain limitations which include the absence of scores that allow for direct assessments of capability or incapability. Instead, it needs to be combined with the clinical observation obtained from the diagnostic process, the psychological evaluation, and the psychiatric and psychosocial history.

Furthermore, it only evaluates the current clinical situation in which there is a need to intervene, by communicating the patient information regarding the disease and the recommended treatment, the risk/benefit ratio, and any treatment alternatives. Thus, the evaluation remains contextualized to that specific situation, without the possibility of generalizing the conclusions, i.e., inferring a definition of the general patient’s capacity to make treatment decisions.

The assessment of general decision-making capacity has significant legal implications. The Italian Civil Code provides for the nullity of legal acts performed by individuals “incapable of understanding or willing” at the time the acts were carried out (Article 428 of the Italian Civil Code). The Italian legal system specifies that “legal capacity”, i.e., the entitlement to rights and duties acquired upon reaching the age of 18, can be lost in cases of “more or less severe infirmities”, “either temporarily or permanently”. In the event of “habitual mental infirmity” and an inability to “provide for one’s own interests”, the person is placed under “interdiction” (Article 414 of the Italian Civil Code) and loses the capacity to act. This means they can no longer perform legal acts such as entering into marriage, managing their own assets, giving valid consent to medical treatment, etc. A “legal guardian” is then appointed to act on behalf and in the exclusive interest of the interdicted individual for their “protection”.

Recognizing the variability of decision-making capacity, both in common legal acts and in the context of informed consent in healthcare, led in 2004 to the introduction of the legal institution of the “Support Administrator” (Article 404 of the Italian Civil Code). This is a legal figure appointed by the Judge in the case of a “person who, due to an infirmity or physical or mental impairment, is unable, even partially or temporarily, to provide for their own interests”. In the decree appointing the Support Administrator, the Judge specifies various areas in which the person will be administered based on their remaining capacities. It is no longer a complete exclusion of legal capacity but rather, a form of support provided in areas where the individual lacks specific capacities. Regarding these capacities, one of the matters under consideration is the ability to give consent in the healthcare context. This necessitates the use of instruments that provide a general indication of this capacity to guide the Judge’s decisions.

Internationally, it is worth noting that the same principles are established by the Convention on the Rights of Persons with Disabilities, adopted by the General Assembly of the United Nations in 2006. Specifically, Article 12 of this Convention calls on member states to take “appropriate measures to provide access by persons with disabilities to the support they may require in exercising their legal capacity”.

As we wanted to test the present evaluation standards of informed competence for people with schizophrenia, in order to make subsequent proposals, we first aimed to perform a pilot study to verify the validity of an evaluation of patients’ competence conducted exclusively through a clinical assessment by clinicians with different backgrounds (psychiatrist, forensic psychiatrist, geriatrician, anesthetist, and medico-legal doctor).

The clinical assessment was then compared to that performed through previously validated scales for assessment of competence and to the appraisal derived from a new scale tested in this study, the “Evaluation of Informed Consent to Treatment” (EICT) scale (see Supplementary material). This is a novel instrument based on a semi-structured interview, designed to address specific challenges encountered in existing tools. Its purpose is to generate conclusions that can be applied across various healthcare and legal contexts by evaluating patients’ general approach to their medical issues. Finally, a neuropsychological evaluation of the main cognitive domains frequently impaired in schizophrenia and involved in competence to give informed consent was performed, to test its contribution to the assessment of patients’ competence to consent.

## 2 Materials and methods

### 2.1 Sample

In the present study, participants diagnosed with schizophrenia (“Sz” group) and regularly followed up at the Santa Lucia Foundation Outpatient Psychiatry Service were consecutively recruited during scheduled visits. To be eligible for potential enrollment in the study, patients had to have a confident diagnosis of schizophrenia based on DSM-5 criteria (American Psychiatric Association, 2013) and be native Italian speakers.

Patients had to be on stable dosages of one or more atypical antipsychotics for at least 6 weeks. Preliminary diagnosis of schizophrenia was made by physicians who usually cared for the patients and knew their medical history. These clinicians were unaware of the purpose of the present study. The diagnosis was subsequently confirmed by an expert clinical researcher through the Structured Clinical Interview for the DSM-5 (SCID-5) (Shankman et al., 2018). Where an agreement on the diagnosis was not reached, further data were collected, and the diagnostic process continued until a shared diagnosis was reached between clinicians and the clinical researcher. Otherwise, patients were not considered for the study.

The inclusion criteria were: i) age between 18 and 65 years, ii) at least 8 years of schooling, iii) general competence to sign an informed consent for inclusion in the present study, as assumed by the fact that the patient was not tutored by a support administrator or a legal custodian.

The general exclusion criteria were: (i) dependence on or abuse of drugs or alcohol (as defined by DSM-5) in the last 5 years, (ii) history of head trauma with loss of consciousness, (iii) neurological disorders, (iv) intellectual disability, (v) a major or mild neurocognitive disorder according to DSM-5 criteria, and (vi) a score at the Mini-Mental State Examination (MMSE) less than 25, according to the normative data of the Italian population (Folstein et al., 1975; Measso et al., 1993).

Patient-specific exclusion criteria were: (i) the evidence of clinical instability within 6 weeks before assessment, including either admission to a psychiatric ward or changes in drug therapy and (ii) comorbidity with other psychiatric disorders.

According to these criteria, 36 patients diagnosed with schizophrenia were selected and approached by the researcher responsible for the study (CD) to provide them with a comprehensive explanation of the study rationale. None of them declined to participate.

From a pool of cognitively fit and neurologically healthy individuals, routinely screened for cognition at the Santa Lucia Foundation Outpatient Centre for Cognitive Deficits and Dementia (“Centro per Disturbi Cognitivi e Demenze,” “CDCD”), who had previously given their consent to be contacted for participation in research studies, 40 individuals were chosen by the same researcher as healthy control subjects (“Hc” group).

Careful one-to-one matching with the included patients with schizophrenia was conducted based on gender and age, and these individuals were approached after their scheduled visit to explain the study procedures. After information, six healthy comparators declined to participate as the proposed visits could not fit into their schedule.

They underwent screening for current or lifetime history of mental disorders based on DSM-5 criteria, utilizing the Structured Clinical Interview for the DSM (SCID), and for Personality Disorders, the SCID-5-PD (Somma et al., 2017). A first-degree relative with a psychosis diagnosis was an additional exclusion criterion for healthy comparators.

### 2.2 Assessment

All participants in the present study underwent the clinical evaluation of competence to consent in the healthcare field and neuropsychological assessment. Psychopathological assessments were conducted for the patient group. The evaluation was performed in more visits, during which trained researchers, independent from the standard care process, tested competence to consent, collected clinical data, and administered all the investigator-rated measures. Socio-demographic and medication-related variables were collected from patients and the clinical records.

#### 2.2.1 Clinical assessment

The age of onset of the disorder was defined as the age at which positive or negative symptoms emerged and was established on the basis of interviews with the patients or with their first-degree family members. When not derivable, the age of onset was established as the age of first hospitalization. Disease duration was defined as the difference between patient age at evaluation and age at onset, in years.

The Positive and Negative Syndrome Scale (PANSS) (Kay et al., 1987), a well-established tool developed for use in schizophrenia, was used to identify the presence and severity of symptoms. For the assessment of the dimensions underlying the symptoms, we used the 5-factor model identified by Wallwork et al. (2012): “positive factor” (sum of items P1, P3, P5, and G9), “negative factor” (sum of items N1, N2, N3, N4, N6, and G7), “disorganization/concreteness factor” (sum of items P2, N5, and G11), “excitation factor” (sum of items P4, P7, G8, and G14) and “depression factor” (sum of items G2, G3, and G6), with a focus on these last three.

The Insight Scale (IS) was used to assess patients’ awareness of their disease, the need for treatment, and the attribution of symptoms (Marková et al., 2003).

The severity of drug-induced extrapyramidal syndromes, akathisia, and dyskinesias was assessed by using, respectively, the Simpson-Angus Scale (SAS) (Simpson and Angus, 1970), the Barnes Akathisia Scale (BARS) (Barnes, 1989) and the Abnormal Involuntary Movement Scale (AIMS) (Guy, 1976).

The daily dosage of antipsychotic treatment was converted to olanzapine equivalents (Leucht et al., 2015).

#### 2.2.2 Evaluation of the competence to give informed consent

##### 2.2.2.1 Clinical interview and global assessment of dimensions underlying competence to consent

The reliability of clinical judgments on competence to consent was tested through unstructured interviews conducted by five expert physicians, specialized in five different disciplines (psychiatry, forensic psychiatry, geriatrics, anesthesia, forensic, and legal medicine).

The choice of medical professionals was based on their specific qualifications to obtain the patients’ consent for diagnostic procedures and treatment in the healthcare setting.

The selection of the five specialists was made considering the medical disciplines with which patients with psychiatric disorders are mostly confronted. The interviewers simulated, individually with each participant, clinical scenarios relevant to the disorders within their respective fields.

To obtain an objective score from each specialist, a GAF scale was adapted to each of the four dimensions investigated (Jones et al., 1995). GAF is a simple and complete tool for evaluating social, occupational, and psychosocial functioning with an overall score ranging from 0 to 100, with higher scores representing better functioning.

Four GAF scales were used in the present study, each adapted for appraising the four underlying dimensions of competence to consent, i.e. comprehension, evaluation, reasoning, and expressing a choice.

##### 2.2.2.2 The Evaluation of Informed Consent to Treatment (EICT) scale

The EICT scale was formulated to investigate how patients normally behave when they have a health problem, evaluating their ability to decide for themselves in a conscious and aware way, free from constraints and suggestions. The scale has been proposed along with a guide for the evaluation, that is a semi-structured interview to be validated during the present study, aimed at assessing patients’ ability to give competent informed consent. The interview includes questions specifically designed for the investigation of the different aspects of patients’ competence dimension:

- to determine the ability to understand information relating to care, some questions investigate the ability to summarize, hierarchize, and integrate them, and to assign meaning to the information through confirming or disproving expectations, and making semantic ineferences.
- in order to assess the capacity to evaluate the significance of data pertaining to a particular scenario, certain inquiries were incorporated concerning the assessment of advantages and disadvantages as well as the associated consequences. Additionally, the ability to actively seek out information and ascertain its reliability was tested, as well as the capacity to evaluate uncertainties. Furthermore, the ability to assess the long-term consequences of one’s own decisions and the impact on others, such as family, friends, and colleagues, was examined.
- in order to assess one’s capacity for coherent reasoning in the decision-making process regarding care, certain inquiries pertained to contemplating the advantages and disadvantages of treatment, the aptitude for logical reasoning, and the awareness of the underlying process that guides one’s choices.
- to determine the ability to express a coherent choice about treatments, some questions were included that investigate volition, i.e., the ability to take initiative voluntarily and to manifest and control behaviors, motivation, and the ability to manage ambivalence.

These four cognitive dimensions underly the ability domains to give competent informed consent identified by literature (Grisso and Appelbaum, 1998; Appelbaum, 2006): Understanding, Evaluating, Reasoning, and Expressing a Choice. Each subscale comprises different items: 4 for Understanding, 7 for Evaluating, 3 for Reasoning, and 3 for Expressing a choice.

Each of the items is associated with an exploratory question in the semi-structured interview; an evaluation is then given on a scale ranging from 0 (absent ability) to 4 points (excellent ability) passing through intermediate scores (1 = doubtful; 2 = sufficient; 3 = good).

Each subscale yields an overall score calculated as the average of the scores obtained in individual items, ranging from 0 to 4. The total scale score is the sum of the scores obtained on the 4 subscales and ranges from 0 to 16.

##### 2.2.2.3 MacArthur Competence Assessment Tool for Treatment (MacCAT-T)

The MacCAT-T was used to assess the ability to consent to treatment. As EICT, it includes a semi-structured interview that investigates the four dimensions of competence to make treatment decisions (Grisso et al., 1997), namely, Understanding, Appreciation, Reasoning, and Expressing a choice. The patient is communicated information regarding one’s clinical condition and which treatment is recommended, its consequences in terms of risks and benefits, and any treatment alternatives with relative risks and benefits. Then the interviewer asks questions to the patient to assess the abilities in the four aforementioned dimensions. Ratings are 2, adequate; 1, partial; and 0, inadequate. The Understanding subscale ranges from 0 to 6, with higher scores indicating better understanding. The Appreciation subscale ranges from 0 to 4, Reasoning from 0 to 8, and Expressing a choice from 0 to 2. At variance with the EICT, the tool assesses the ability to give informed consent in the context of a single medical treatment. Moreover, although some MacCAT-T cut-offs have been proposed (Kim et al., 2011; Mandarelli et al., 2014; Lepping et al., 2015), the MacCAT-T manual does not provide a definite threshold, nor a total score useful to define when a patient should be considered competent or incompetent.

In the present study, we used as a clinical situation the simulation of the choice between two different treatments, with relative risks and benefits, for the clinical condition affecting the patients (schizophrenia).

##### 2.2.2.4 Structured Interview for Competency and Incompetency Assessment Testing and Raniking Inventory-Revised (SICIATRI-R)

The scale is based on a structured interview comprising 12 items, also investigating the 4 fundamental dimensions involved in the ability to give informed consent (Kitamura and Kitamura, 2011). Each item is provided with multiple response alternatives and the most appropriate response alternative has to be filled in the summary sheet. Each response is then classified on a 5-point scale, ranging from 1 (inadequate performance) to 5 (adequate performance). For each item, the authors provide the questions, the explanation, and the “anchor points” with the definitions. After completing the interview, the interviewer should make a ranking according to the attached Ranking Inventory for Competency, to classify the patient’s competency into five categories, from level 0 (complete incompetency) to level 4 (complete competency).

#### 2.2.3 Neuropsychological evaluation

The Mini-Mental State Examination (MMSE) (Folstein et al., 1975) was used as a general screening test for cognitive function. It is constituted of 30 items that refer to different cognitive areas: orientation in time and space, memory, attention and calculation, memory, language, and constructive praxis. The total score is between 0 and 30 points. Conversely, the dimensions constituting the ability to give informed consent were assessed through *ad hoc* selected neuropsychological tests.

The Rey Auditory Verbal Learning Test (RAVLT) (Rey, 1964) is a well-recognized measure of a person’s ability to encode, combine, store, and recall verbal information in different stages of immediate (RAVLT Immediate, RAVLT-I) and delayed memory (RAVLT Delayed, RAVLT-D, after 30 min of interpolated testing).

The Raven’s Coloured Progressive Matrices Test (Raven-CPM) (Raven et al., 1998) measures the main components of general intelligence and in particular logical-inductive reasoning to solve problems that cannot be resolved based on previous knowledge (Cattell, 1987). Evaluating the ability to understand relationships between abstract figural elements is considered a cultural-free test (Raven and Raven, 2008).

Visuospatial processing and working memory were measured with Trail Making Test A and B (TMT A and TMT B), respectively assessing simple attention (TMT A) and set-shifting (TMT B) (Reitan, 1958). A cut-off of 94 s for TMT A and 283 s for TMT B is considered normative for the Italian general population (Giovagnoli et al., 1996).

Semantic and Phonemic verbal fluency tasks (Benton, 1968) were included to test executive functioning, speed, attention, as well as access to the mental lexicon. Semantic fluency was investigated by asking the participant to generate as many animal names as possible in 60 s. In the phonemic fluency task, participants were asked to generate as many words as possible starting with the letter “F” in 60 s excluding proper nouns.

The understanding dimension was assessed with the MT battery which measures the participants’ ability to find appropriate information in the text to answer a series of comprehension questions, i.e., in order to assess subjects’ understanding regardless of their ability to decode and recall the text (Cornoldi and Oakhill, 1996; Cornoldi et al., 2010).

The following tasks of the MT battery were administered: Text A for the evaluation of the ability to infer the meaning of words based on the context (lexical inference); Text C for the evaluation of the ability to correct inconsistencies and suspend hypotheses, using subsequent information for understanding; Text E for the evaluation of the ability to gain important elements and derive the “core” meaning.

For the assessment of the Evaluation dimension, the Zoo Map (ZM) subtest from the Behavioral Assessment of Dysexecutive Syndrome (BADS) battery was used (Wilson et al., 1996). It is a test in which the subject has to plot or follow a route through a map that does not contravene a set of rules. It assesses the ability to formulate and implement a plan (spontaneous planning skills) and to follow a pre-formulated plan (guided planning skills). Penalties are imposed for rule breaking and lack of speed. This test was also useful to assess the ability to Expressing a choice.

The Reasoning was assessed through the following tests:

- Meta-Wisconsin Cart Sorting Test Categories (Meta-WCST CAT), Perseverative Errors (Meta-WCST PE), and Levels of Confidence (Meta-WCST LC) (Koren et al., 2005), a modified version of WCST used for the evaluation of metacognitive abilities in reasoning and problem-solving tasks. Subjects are asked to rate their degree of certainty (Levels of Confidence) in the categorization task before feedback is given from the examiner. The correct Categories, the Confidence Level, and the Perseverative Errors were scored.
- Verbal judgments (Spinnler and Tognoni, 1987), a test that evaluates verbal reasoning.

Finally, for the evaluation of the Expression of a choice, the following tests were administered:

- The Dysexecutive Questionnaire (DEX) from the BADS battery; is a 20-item self-assessment questionnaire with a Likert-type scale that investigates executive functioning in activities of daily living.
- The Decision Self-Efficacy Scale (DSES) (O’Connor, 1995), a self-assessment questionnaire that elicits patients’ appraisal of their abilities to engage in the task of obtaining information about treatment options, expressing their concerns and views, and making an informed choice. It is based on 11 items with a five-point response scale ranging from 0 (not at all confident) to 4 (very confident).
- The Gudjonsson Suggestibility Scales-2 (GSS-2) is one of two scales developed by Gudjonsson to test interrogative suggestibility and to be used as forensic tools to help assess the reliability of confessions. The scales also help identify those individuals who may be particularly vulnerable to the pressures associated with interrogations and who, as a result, may require special attention during questioning. The narrative content of GSS 2 is less complex than that of GSS 1 and, for this reason, GSS 2 is more commonly used for people with cognitive impairments.

### 2.3 Statistical analysis

The socio-demographic characteristics of the patients and control groups were analyzed. As a priority, an analysis of the correlation between the five referring clinicians in the evaluation of the same subjects was performed, calculating the Pearson correlation coefficient (r) between the scores of the GAF scales. The levels of convergence or divergence between clinical judgments and the EICT subscales were measured by calculating the Pearson correlation coefficient (r) between the scores obtained on the four subscales of the EICT and the scores of the GAF scales for the same four dimensions.

The “Sz” group and the control subjects (“Hc” group) were then compared for clinical and neuropsychological variables based on the ability to give informed consent to treatment measured by the three scales using Student’s t-test and Levene’s test for the equality of variances.

The same analysis was conducted to delineate the different clinical and neuropsychological characteristics of schizophrenic patients able (“Sz-A”) and not able (“Sz-NA”) to give informed consent to treatment, as defined by their EICT score.

Given the gender imbalance, a Student’s *t*-test comparison between male and female patients’ scores was performed for EICT, MacCAT-T, and SICIATRI-R, other than for clinical and neuropsychological tests.

All statistical analyses were performed using IBM® SPSS® Statistics version 29; *p*-values <0.05 (two-tailed) were considered significant.

### 2.4 Reliability and validity analysis

The interview associated with the EICT scale was initially administered to five pilot patients, but it was not necessary to make substantial changes. Reliability and internal consistency of the scale were calculated using Cronbach’s alpha coefficient (Cronbach, 1951) both for the entire scale and the subscales (Understanding, Evaluation, Reasoning, and Expressing a choice).

A single item-total scale correlation and, for the items of the specific sub-scale, a single item-subscale correlation, using Pearson correlation coefficient (r) (Pearson, 1909), were performed, to evaluate the reliability and relevance of each item.

In order to test the validity of the scale using external criteria, the convergent validity was evaluated through the comparison with tools already validated for the examination of the same construct, i.e., the decision-making competence. Therefore, the Pearson correlation coefficient (r) was calculated between the subscales of the EICT and the corresponding subscales of the MacCAT-T and between the total score of the first with that of the SICIATRI-R.

Similar analyses were conducted to evaluate the correlation between the results obtained on the four subscales of the EICT and the scores obtained on the neuropsychological tests associated *a priori* to the sub-areas and used to individually investigate the functions believed to underlie the cognitive domains connected with decision-making ability.

Finally, a cut-off score was calculated for each of the four subscales by identifying the threshold score which identifies the probability that a subject belongs to one or the other of the two distributions.

We adopted the following formulation for the cut-off = (DS_Sz*M_Sz + DS_Hc*M_Hc) / (DS_Sz + DS_Hc), where: DS_Sz = standard deviation of the Sz sample; M_Sz = sample mean of the Sz sample; DS_Hc = standard deviation of the Hc sample; M_Hc = mean of the Hc sample.

The EICT scale total score cut-off was calculated as the sum of the cut-off scores of the four subscales, each of which is essential for competence, considering that subthreshold performance on a single subscale predicts incomplete competence. As said, based on the EICT scale cut-off score patients with schizophrenia were further divided into able (“Sz-A”) or not-able (“Sz-NA”) to consent.

## 3 Results

### 3.1 Sample Characteristics

Thirty-six subjects diagnosed with schizophrenia and thirty-four control subjects were included in the study (Table 1). Two subjects were subsequently excluded from the group of patients with schizophrenia as they developed clinical instability during the study and no longer fulfilled the inclusion criteria.

**Table 1:** “Sz” and “Hc” groups demographic and clinical characteristics.

Variable	“Sz” (*n* = 34) mean ± SD	“Hc” (*n* = 34) mean ± SD
Age (years)	41.85 ± 9.74	38.59 ± 9.5
Gender M:F *n*. (%)	25:9 (73.5:26.5)	25:9 (73.5:26.5)
Education (years)	12.59 ± 2.74	15.24 ± 3.03
Age of onset (years)	23.16 ± 6.19	N/A
Duration of illness (years)	18.22 ± 9.38	N/A
Olanzapine equivalents (years)	18.21 ± 30.18	N/A
PANSS positive	14.79 ± 5.16	N/A
PANSS negative	21.59 ± 6.26	N/A
PANSS general	34.68 ± 9.51	N/A
PANSS total	71.06 ± 17.14	N/A
PANSS positive factor^a^	8.82 ± 4.25	N/A
PANSS negative factor^a^	18.50 ± 5.74	N/A
PANSS disorganized/concrete factor^a^	7.12 ± 3.40	N/A
PANSS excited factor^a^	6.38 ± 3.02	N/A
PANSS depressed factor^a^	5.97 ± 2.44	N/A
IS	13.25 ± 5.65	N/A
SAS	3.66 ± 2.84	N/A
AIMS	0.37 ± 0.76	N/A
BARS	0.43 ± 0.728	N/A

Significant *p*-values highlighted in bold.

a Wallwork’s factors for PANSS.

The final sample was composed of 68 subjects (male = 50, female = 18), divided into 34 for the “Sz” group (male = 25, female = 9) and 34 for the “Hc” group (male = 25, female = 9).

The socio-demographic data of patients, as well as the clinical data of the latter, i.e., the components of PANSS and the five dimensions of PANSS identified by Wallwork, IS, SAS, AIMS, BARS, the age of onset, the duration of the disease, and olanzapine equivalents of the drug therapy, are reported in Table 1. Education level was the only variable that could not be matched because control subjects had a higher level (years 15.24 ± SD 3.03) than patients with schizophrenia (years 12.59 ± SD 2.74; *p* < 0.001) given that severe mental health disorders, particularly if present early in life, are associated with disruption in schooling (Crossley et al., 2022).

### 3.2 Evaluation by five referring clinicians

The analysis of the correlations indices of Pearson’s (*r*) between the clinical judgments provided by the five specialists involved in the study (scores on the GAF scales on the four areas), revealed:

- For the GAF-Understanding, a correlation ranging from 0.54 (highest correlation between the psychiatrist and the anesthetist) and 0.09 (lowest correlation between the forensic psychiatrist and the medico-legal doctor);
- For the GAF-Evaluation, a correlation ranging from 0.62 (highest correlation between the psychiatrist and the geriatrician) and 0.14 (lowest correlation between the geriatrician and the medico-legal doctor);
- For the GAF-Reasoning, a correlation ranging from 0.65 (highest correlation between the psychiatrist and the geriatrician) and 0.22 (lowest correlation between the forensic psychiatrist and the medico-legal doctor);
- For the GAF-Expressing a choice, a correlation ranging from 0.53 (highest correlation between the geriatrician and the anesthetist) and − 0.01 (lowest correlation between the forensic psychiatrist and the medico-legal doctor).

The correlations between the EICT subscales and the respective GAF scales elaborated by the five clinicians were also highly variable, in particular:

- For EICT Understanding the correlation ranged from 0.6 with the GAF elaborated by the forensic psychiatrist and 0.04 with that by the medico-legal doctor (Table 2).
- For EICT Evaluating the correlation ranged from 0.69 with the GAF elaborated by the forensic psychiatrist and − 0.06 with that by the medico-legal doctor (Table 3).
- For EICT Reasoning the correlation ranged from 0.6 with the GAF elaborated by the psychiatrist and the geriatrician, and 0.3 with that by the medico-legal doctor (Table 4).
- For EICT Expressing a choice the correlation ranged from 0.55 with the GAF elaborated by the forensic psychiatrist and − 0.17 with that by the medico-legal doctor (Table 5).

**Table 2:** EICT understanding and GAF understanding correlation.

Specialist	Correlation *r* di Pearson	*p*-value	95% lower	95% upper
Medico-Legal Doctor	0.04	0.8851	−0.414	0.47
Anesthesist	0.27	0.2456	−0.191	0.639
Psychiatrist	0.51	0.021	0.084	0.776
Geriatrician	0.57	0.0083	0.163	0.806
Forensic Psychiatrist	0.6	0.0046	0.208	0.822

**Table 3:** EICT evaluating and GAF evaluating correlation.

Specialist	Correlation *r* di Pearson	*p*-value	95% lower	95% upper
Medico-Legal Doctor	−0.06	0.7981	−0.491	0.391
Anesthesist	0.37	0.1147	−0.093	0.695
Psychiatrist	0.47	0.0379	0.028	0.753
Geriatrician	0.53	0.0157	0.11	0.786
Forensic Psychiatrist	0.69	0.0004	0.36	0.869

**Table 4:** EICT reasoning and GAF reasoning correlation.

Specialist	Correlation *r* di Pearson	*p*-value	95% lower	95% upper
Medico-Legal Doctor	0.3	0.2918	−0.216	0.624
Anesthesist	0.3	0.2468	−0.192	0.639
Psychiatrist	0.5	0.0267	0.062	0.767
Geriatrician	0.6	0.004	0.22	0.826
Forensic Psychiatrist	0.6	0.0023	0.257	0.838

**Table 5:** EICT Expressing a choice and GAF expressing a choice correlation.

Specialist	Correlation *r* di Pearson	*p*-value	95% lower	95% upper
Medico-Legal Doctor	−0.17	0.4862	−0.568	0.297
Anesthesist	0.32	0.1653	−0.138	0.671
Psychiatrist	0.37	0.1108	−0.088	0.697
Geriatrician	0.42	0.0628	−0.024	0.729
Forensic Psychiatrist	0.55	0.0117	0.135	0.796

### 3.3 Between groups comparison

Group comparisons showed overall significant differences (the “Hc” group performed better overall) in the ability to give informed consent to treatment, as measured by the three scales (EICT, MacCAT-T, and SICIATRI -R), and associated neuropsychological test performance, except for the RAVLT-D (*p* = 0.068) (Table 6).

**Table 6:** Comparison between “Hc” and “Sz” groups for competence and neuropsychological characteristics.

Variable	Hc mean ± SD	Sz mean ± SD	*t*-test	*p*-value
EICT Total score	15.58 (±1.41)	6.61 (±3.17)	15.090*	<0.001
Sub. EICT Und.	3.87 (±0.39)	1.55 (±0.84)	14.469*	<0.001
Sub. EICT Eval.	3.91 (±0.36)	1.67 (±0.84)	14.196*	<0.001
Sub. EICT Reas.	3.89 (±0.39)	1.76 (±0.97)	11.905*	<0.001
Sub. EICT Exp.	3.91 (±0.38)	1.63 (±0.84)	14.508*	<0.001
SICIATRI-R	4 (±0)	2.50 (±1.81)	4.824*	<0.001
Sub. MacCAT-T Und.	5.90 (±0.33)	4.05(±1.44)	7.265*	<0.001
Sub. MacCAT-T Eval. (Val Tot)	4 (±0)	2.74 (±1.35)	5.441*	<0.001
Sub. MacCAT-T Reas. (Rag Tot)	7.94 (±0.34)	4.37 (±2.18)	9.447*	<0.001
Sub. MacCAT-T Exp. (Ded Cons)	1.971 (±0.17)	1.19 (±0.77)	5.700*	<0.001
RAVLT-I	46.13 (±5.74)	31.82 (±12.01)	6.265*	<0.001
**RAVLT-D**	9.56 (±2.12)	6.79 (±8.324)	1.881*	**0.068**
Raven-CPM	29.62 (±2.69)	27.34 (5.33±)	3.781*	0.030
TMT-A (sec)	53.29 (±12.04)	81.61 (±39.1)	−3.981*	<0.001
TMT-B (sec)	102.74 (± 26.91)	187.64 (±99.75)	−4.725*	<0.001
Semantic Verbal Fluency	25.91 (±5.34)	15.09 (±4.47)	9.054	<0.001
Phonemic verbal fluency	44.47 (±10.54)	28.24 (±9.55)	6.655	<0.001
Meta-WCST-Cat.	3.00 (±0)	2.44 (±0.96)	3.396*	0.002
Meta-WCST-PE	2.68 (±1.09)	6.32 (±6.23)	−3.361*	0.002
Meta-WCST-LC	91.57(±10.37)	76.40(±24.02)	3.380*	0.002
GSS-2	7.65 (±4.55)	14.03 (±7.82)	−4.111*	<0.001
Verbal judgments	54.21 (±5.09)	41.97 (±9.22)	6.770*	<0.001
Zoo Map	12.12 (±3.85)	5.56 (±6.13)	5.282	<0.001
MT Text A	8.37 (±1.35)	6.41 (±2.30)	3.563*	0.001
MT Text C	8.24 (±1.74)	5.31 (±2.47)	5.527*	<0.001
MT Text E	5.91 (±1.54)	4.42 (±1.92)	3.498*	0.001
MMSE	29.62(±0.60)	28.26 (±1.35)	5.317*	<0.001
DEX	10.94 (±8.64)	28.21 (±18.25)	−4.985*	<0.001
DSES	90.10 (±16.35)	67.65 (±20.59)	4.980	<0.001

* Levene’s test *p*-value < 0.05. *p*-values above the threshold for statistical significance are highlighted in bold.

As for the comparison between the clinical and neuropsychological characteristics of schizophrenic patients who were able (“Sz-A”, *n* = 11) or not able (“Sz-NA”, *n* = 23) to give informed consent to treatment, significant differences emerged for the Phonemic verbal fluency task (*p* = 0.038), Verbal judgments (*p* = 0.048), EICT Und. (*p* < 0.001), EICT Eval. (*p* < 0.001), EICT Reas. (*p* < 0.001), EICT Exp (*p* < 0.001), MacCAT-T Und. Tot (*p* = 0.01), MacCAT-T Eval. (*p* < 0.001), MacCAT-T Reas. (*p* < 0.001), MacCAT-T Exp. (*p* = 0.004), SICIATRI-R (*p* < 0.001) (Supplementary Table S1).

No gender differences in the patients’ group were found with regard to the scales measuring the competence (EICT-T, MacCAT-T, and SICIATRI-R), nor to the associated neuropsychological tests, except for the RAVLT-I (*p* < 0.05), in which females performed better (Supplementary Table S2).

### 3.4 Reliability and validity

Cronbach’s alphas of each subscale and the overall EICT score exhibited excellent internal consistency: EICT Understanding α = 0.97, EICT Evaluation α = 0.98, EICT Reasoning α = 0.97, EICT Expression of a choice α = 0.96; EICT total score α = 0.99.

The Item Analysis, by measuring the correlation between each item of the EICT and the total score, with Pearson’s (r) coefficient, was excellent for all items of all subscales, ranging from 0.86 to 0.98 (Supplementary Table S3).

The convergent validity was evaluated through the comparison with other assessment tools, exhibiting an excellent correlation with the already existing scales that evaluate the same construct.

Specifically, the Pearson correlation coefficient (r) between the EICT subscales and the corresponding MacCAT-T subscales was for Understanding *r* = 0.87 (*p* < 0.0001); Evaluating *r* = 0.61 (p < 0.0001); Reasoning *r* = 0.92 (*p* < 0.0001); Expressing a choice *r* = 0.57 (*p* < 0.0001). Also, EICT and SICIATRI-R total scores correlated, with an *r* = 0.72 (*p* < 0.0001).

The subscales of the EICT and the related neuropsychological tests, *a priori* associated with each subscale, also exhibited good correlations:

- For EICT Understanding and MT tasks, the correlation with Text A was *r* = 0.66 (*p* < 0.0001), with Text C, *r* = 0.73 (*p* < 0.0001), and with Text E *r* = 0.43 (*p* = 0.0018);
- For EICT Evaluating and Zoo Map, *r* = 0.49 (*p* = 0.0002);
- For EICT Reasoning and Meta-WCST Categories *r* = 0.51 (*p* < 0.0001); Verbal judgments *r* = 0.72 (*p* < 0.0001);
- For EICT Expressing a choice and Meta-WCST Levels of Confidence *r* = 0.30 (*p* = 0.0336); Zoo Map *r* = 0.50 (*p* = 0.0001).

The Dex, DSES and GSS-2 questionnaires correlated with all EICT subscales:

- For EICT Understanding and Dex *r* = −0.60 (*p* < 0.0001), DSES *r* = 0.43 (*p* = 0.0017), GSS-2 *r* = −0.50 (*p* = 0.0002);
- For EICT Evaluation and Dex *r* = −0.53 (*p* < 0.0001), DSES *r* = 0.40 (*p* = 0.0031), GSS-2 *r* = −0.42 (*p* = 0.0022);
- For EICT Reasoning and Dex *r* = −0.58(*p* < 0.0001), DSES *r* = 0.41 (*p* = 0.0027), GSS-2 (*r* = −0.47; *p* = 0.0005);
- For EICT Expressing a choice and Dex *r* = −0.56 (*p* < 0.0001), DSES *r* = 0.50 (*p* = 0.0002), GSS-2 *r* = −0.42 (*p* = 0.0022).

The cut-off scores for each of the four subscales resulted to be 2 for each subscale, in line with the theoretical premises. The cut-off for the entire scale was found to be 8.

## 4 Discussion

In our study, the initial goal was to assess the reliability of evaluating patients’ capacity to provide consent, relying solely on clinical judgment, even when expressed by attending physicians.

The evaluation of the clinical assessments carried out by five separate medical experts (including a psychiatrist, forensic psychiatrist, geriatrician, anesthesiologist, and medico-legal doctor) revealed the shortcomings of assessing the capacity to provide informed consent without standardized instruments. This was demonstrated by the significant variation among clinicians in determining competence.

Analogous variability emerged for the correlation between the judgments provided by clinicians through the GAF scales, adapted to the four dimensions of the competence investigated, and the subscales of the Evaluation of Informed Consent to Treatment (EICT) scale.

The latter is a new tool designed for the assessment of the general competence to consent of patients with psychiatric disorders. It was tested in the current study and may prove useful in guiding clinical and legal decisions regarding the capacity of patients to make decisions in the healthcare field.

In general, our results emphasize to physicians the importance of not relying exclusively on their own clinical expertise and experience.

It is conceivable that clinicians may place too much emphasis on the diagnosis, potentially underestimating the true capabilities of the patients (Tomoda et al., 1997). Moreover, aspects on which they can rely include psychopathology and patients’ awareness of their mental disorders.

It is recognized that the latter aspect can be compromised in patients with severe mental illnesses (SMI), especially in schizophrenia spectrum and other psychotic disorders, as well as in bipolar disorders, rendering them incapable of giving informed consent (Ruissen et al., 2012).

Generally, clinicians tend to posit that awareness of illness and its implications is diminished in SMI, particularly during acute phases of disorders, while it may improve during the remission phases, influencing fluctuations in competence (Chakraborty and Basu, 2010).

Nevertheless, a meta-analysis revealed that only 3–7% of the variability in overall insight (including awareness of the mental disorder, awareness of the social consequences of disorder, awareness of the need for treatment, awareness of symptoms, and attribution of symptoms to the disorder) could be attributed to the severity of symptoms, indicating a limited impact on its overall degree (Mintz et al., 2003). Moreover, it has been observed that non-psychotic patients with adequate overall insight can demonstrate incompetence in a significant number of cases (Ruissen et al., 2012).

Hence, the risk for clinicians is to emphasize patients’ clinical stability during evaluations, potentially underestimating their cognitive difficulties that impact complex decision-making (Appelbaum and Roth, 1982).

Considering these issues, the importance of assessment tools becomes evident as they provide healthcare professionals with information regarding an individual’s capacities.

We also tested the psychometric characteristics and clinical utility of the EICT scale, *ad-hoc* constructed to investigate the ability to give informed consent to treatment and modeled on the already existing MacCAT-T scale (Grisso et al., 1997).

Overall, various scientific contributions have identified four fundamental cognitive dimensions (Understanding, Evaluating, Reasoning, and Expressing a choice) that underlie the ability to decide on issues related to one’s healthcare, namely the diagnostic-therapeutic process, as well as participation in scientific research (Appelbaum and Grisso, 1988; Grisso and Appelbaum, 1998; Appelbaum, 2006; Dunn et al., 2006; Gupta and Kharawala, 2012).

In the construction of this scale, we have considered the fundamental importance of these four dimensions, evaluating them separately.

A deficiency in any of these four dimensions is sufficient to compromise the entire competence, as each equally influences the ability to provide informed consent, particularly regarding the decision-making process.

For instance, individuals who are aware of their mental disorder but struggle to utilize this awareness for evaluating implications, considering alternative treatments, engaging in coherent reasoning about options and consequences, or lack interest in taking initiatives, cannot be deemed fully competent.

Referring to the proposed scale (with the related interview), statistical analyses demonstrated that the EICT scale has satisfactory general reliability.

For a scale to effectively measure a construct, the items should explore its different aspects and therefore be consistent with each other; if this coherence is lacking, it is likely that they investigate different areas, therefore failing to measure the phenomenon of interest.

The high internal consistency of the EICT scale indicates that the items effectively explore various facets of general competence to consent. The Item Analysis also demonstrates that they collectively measure a single latent factor. In terms of validity, our findings suggest that the proposed tool effectively measures the construct for which it was designed.

Additionally, we found a good correlation between the EICT scale total score and that of the SICIATRI-R, as well as its four subscales and the ones of the MacCAT-T, although these other scales measure the four core dimensions of competence based on different assumptions.

Both the MacCAT-T and the SICIATRI-R evaluate competence by considering the patients’ current clinical situation and remaining contextualized within that specific scenario. So, it is not possible to generalize the conclusions beyond the given context.

The EICT scale, therefore, has been constructed with the aim of evaluating patients’ ability to make decisions concerning their healthcare in general, not referring to a specific situation.

This evaluation is based on a semi-structured interview consisting of a series of questions for the investigation of the different aspects of each dimension.

For the assessment of the Understanding of information relating to own healthcare, questions are asked that investigate the ability to hierarchize and integrate information, elaborate a meaning through the confirmation and disconfirmation of expectations, and make semantic inferences.

The ability to Evaluate the meaning of information relating to one’s clinical condition is then investigated through questions on the pros and cons of choices, on the active search for information, on weighing its source, considering uncertainty, the implications of one’s choices on others (relatives, co-workers) and their consequences over time.

The ability to Reason about the care decision-making process is investigated through questions related to thinking about the pros and cons, logical reasoning, and awareness of the process leading to the choice.

Finally, the ability to Express a choice on treatment is evaluated through questions that investigate volition, i.e., the ability to voluntarily take initiative and to manifest and control behaviors, the motivation, and the management of ambivalence.

The interview aims to evaluate the general approach of patients toward their medical problems and the conclusions could therefore be generalized.

However, as said, the competence of patients with psychiatric disorders can fluctuate over time, based on the phase of the disease and the predominant symptoms (for example it can be compromised by an acute phase as a manic state, or by the presence of delusions and hallucinations in psychotic disorders) (Palmer et al., 2004; Sturman, 2005; Dawson and Szmukler, 2006; Okai et al., 2007; Owen et al., 2008). Therefore, the conclusions of an assessment of competence should always be updated and integrated with clinical evaluation.

The analysis of the correlation between the EICT subscales and the neuropsychological tests showed a good validity of the scale in measuring the related constructs.

The scales evaluating decision-making self-efficacy, the role of executive functions in daily living activities, and suggestibility correlate transversely with all the dimensions that constitute competence. So, the ability to make conscious decisions in daily life activities and organize oneself, to plan, to be flexible, to solve problems, and to self-regulate (measured by the scales of decision-making self-efficacy and executive functions in daily life) also positively affects competence in healthcare decisions. On the other hand, high suggestibility negatively affects the ability to make autonomous decisions, as subjects are easily influenced, in general, by the opinion of others, and, in healthcare, by their referring doctor, thus failing the fundamental principle of freedom and autonomy of choice.

It should be emphasized that relying on the referring doctor as the main source of information is certainly a correct choice for the patient and denotes a good capacity for discernment. However, it is at the same time important to retain the ability to consider treatment alternatives, to doubt, to ask the doctor for clarification, to critically judge the situation, and to correctly evaluate the meaning of the information.

Actually, from the analysis of the psychopathological characteristics of schizophrenic patients, able and not able to consent, no clinical differences emerged between the two groups, while significant differences were found for phonemic verbal fluency and verbal judgments.

Conversely, all three scales used for the evaluation of competence, as well as the subscales, reached statistical significance in the discrimination between the two groups.

Therefore, a tool to evaluate the ability to give informed consent in the healthcare setting is of primary importance, given the specific capacity for discernment.

The EICT scale, moreover, differently from the MacCAT-T, provides cut-off scores to discriminate the ability to give informed consent. A score below 2 on a subscale suggests insufficient competence, considering that all four dimensions contribute equally to its determination.

The overall score is determined by the sum of the scores for the four subscales; therefore, it must necessarily be equal to or greater than 8.

A further strength is given by the interview that accompanies the EICT, which aims to facilitate its administration, providing guiding questions adaptable to the situation and context.

It is also brief, with a duration ranging between 20 and 30 min, and can be adopted during the clinical interview.

Finally, it is important to emphasize that, despite the usefulness of assessment tools, they do not saturate the evaluation of patients’ capacity, which also relies on comprehensive clinical assessments and the gathering of information from their caregivers.

### 4.1 Limitations

Despite the findings of our study, however, some limitations have to be considered. Given the complexity of the selection and assessment procedures, this was a pilot study involving a limited number of researchers, clinicians, patients, and controls, aiming at exploring preliminary results to engage further resources. A limitation consists in the fact that the assessment of consent by the five clinicians involved did not have a re-test by another five different clinicians, therefore the evaluations are affected by the specificity of the individual examiners. Also, the small sample size of patients and controls limits the generalizability of our findings and could be associated with type II statistical errors. In addition, the number of patients with schizophrenia able to give informed consent to treatment was about half of those unable, resulting in an imbalance.

Furthermore, confirmation of the results for different types of clinical settings is required.

An extension of the study, possibly with a multicenter design, will therefore be necessary, to involve more researchers, increase the sample, and evaluate additional clinical populations, such as patients with other psychiatric disorders.

Finally, our study relied on quantitative data, and the narrative perspectives of patients were not evaluated. The integration of qualitative data from patients regarding their experience in the consent process could provide valuable insights and serve as a useful implementational tool for further development in the future phases of the study.

## 5 Conclusion

In conclusion, the evidence reported suggests primarily that clinical judgments are unreliable in correctly evaluating the competence to consent for people with a severe mental illness such as schizophrenia.

Moreover, despite the large number of neuropsychological tasks investigated in our study, no solid evidence emerged about tests allowing a valid differentiation between competent and non-competent subjects, nor for their psychopathological characteristics.

On the other hand, tools specifically designed to assess competence have demonstrated an excellent discriminating capacity. In the present study, we aimed to validate a new scale with different characteristics compared to the existing assessment tools. Therefore, we proposed the EICT scale, characterized by an interview as a guide, a cut-off for the judgment on competence, and questions aiming at evaluating the patients’ ability to make decisions concerning their healthcare in a general way, not referring to a specific situation.

Given the reliability of the EICT scale, it could be suggested its use in multiple specialistic healthcare settings, where the evaluation of the real competence of patients with psychiatric disorders could be necessary.

An accurate assessment, with the support of standardized tools, is essential to avoid errors that, in the delicate field of informed consent in healthcare settings, could have medico-legal relevance.

Furthermore, the EICT scale could serve as a valuable tool in the context of legal decisions regarding whether and to what extent administrators should support individuals with disabilities.

## Data availability statement

The raw data supporting the conclusions of this article will be made available by the authors, without undue reservation.

## Ethics statement

The studies involving humans were approved by Ethics Committee of the Santa Lucia Foundation of Rome. The studies were conducted in accordance with the local legislation and institutional requirements. The participants provided their written informed consent to participate in this study.

## Author contributions

NDF: Conceptualization, Formal analysis, Investigation, Writing – review & editing. DM: Conceptualization, Writing – original draft. FeP: Formal analysis, Investigation, Project administration, Supervision, Writing – review & editing. FaP: Formal analysis, Investigation, Project administration, Supervision, Writing – review & editing. NB: Project administration, Supervision, Writing – review & editing. GD: Methodology, Writing – review & editing. FD: Methodology, Writing – original draft. PF: Project administration, Supervision, Writing – review & editing. VF: Project administration, Supervision, Writing – review & editing. SF: Formal analysis, Investigation, Project administration, Supervision, Validation, Writing – review & editing. GS: Formal analysis, Investigation, Project administration, Supervision, Validation, Writing – review & editing. CD: Formal analysis, Investigation, Methodology, Validation, Writing – original draft.

## Glossary

Hc: healthy control subjects
Sz: subjects with schizophrenia
Sz_A: subjects with schizophrenia able to consent
Sz_NA: subjects with schizophrenia not able to consent
PANSS: Positive and Negative Syndrome Scale
IS: Insight Scale
SAS: Simpson Angus Scale
AIMS: Abnormal Involuntary Movement Scale
BARS: Barnes Akathisia Scale
EICT: Evaluation of Informed Consent to Treatment Scale
Sub. EICT Und.: Subscale EICT Understanding
Sub. EICT Eval: Subscale EICT Evaluating
Sub. EICT Reas.: Subscale EICT Reasoning
Sub. EICT Exp.: Subscale EICT Expressing a choice
SICIATRI-R: Structured Interview for Competency and Incompetency Assessment Testing and Ranking Inventory-Revised
MacCAT-T: MacArthur Competence Assessment Tool-Treatment
Sub. MacCAT-T Und.: Subscale MacCAT Understanding
Sub. MacCAT-T App.: Subscale MacCAT Appreciation
Sub. MacCAT-T Reas.: Subscale MacCAT Reasoning
Sub. MacCAT-T Exp.: Subscale MacCAT Expressing a choice
RAVLT-I: Rey Auditory Verbal Learning Test- Immediate Recall
RAVLT-D: Rey Auditory Verbal Learning Test- Delayed Recall
Raven-CPM: Raven’s Coloured Progressive Matrices Test
TMT-A: Trail Making Test A
TMT-B: Trail Making Test B
Meta-WCST-Cat.: Meta-Wisconsin Cart Sorting Test-Categories
Meta-WCST-PE: Meta-Wisconsin Cart Sorting Test-Perseverative Errors
Meta-WCST-LC: Meta-Wisconsin Cart Sorting Test- Levels of Confidence
GSS-2: Gudjonsson Suggestibility Scales-2
MMSE: Mini-Mental State Examination
DEX: Dysexecutive Questionnaire
DSES: Decision Self-Efficacy Scale
GAF: Global Assessment of Functioning
SD: Standard Deviation
df: degrees of freedom
