# Supplementary material for: Reliability of clinical judgment for evaluation of informed consent in mental health settings and the validation of the Evaluation of Informed Consent to Treatment (EICT) scale
Source: Front Psychol. 2024 Mar 19;15:1309909. doi: 10.3389/fpsyg.2024.1309909 (PMC10986368; doi:10.3389/fpsyg.2024.1309909)
Supplement: Supplementary file 1 [file Data_Sheet_1.docx]

Supplementary Material

**Reliability of clinical judgment for evaluation of informed consent in mental health settings and the validation of the Evaluation of Informed Consent to Treatment (EICT) scale**

**Clinical Interview for the Evaluation of Informed Consent to Treatment (EICT) Scale**

Interviewer: for instance, "We will spend the next 20-30 minutes discussing some matters related to your health and your approach to medical issues. We will refer to how you generally behave when faced with such types of problems".

*In quotes, some examples of communication that the interviewer can use during the interview.

**Information on Understanding**

1. "Can you start by telling me about the last time you visited the doctor? Why did you go? What did the doctor tell you? Do you think you understood everything that was said to you?". (Ability to summarize and hierarchize information). If yes, investigate about what the patient understood (e.g., ask about the condition, potential risks, available treatments, etc.) and then proceed to question number 6. If after the exploration the patient is considered unreliable, proceed with question number 2.

2. "What difficulties did you have in understanding?".

3. "Did you encounter difficulties with the terminology used?".

4. "Did you understand what the long-term consequences of your condition/problem could be?".

5. "Did you have difficulty understanding the treatments/medications proposed to you?".

6. "Could you tell me what, in your opinion, were the most relevant pieces of information that the doctor gave you?" (Ability to summarize and hierarchize information).

7. "In your opinion, is there a connection between the condition and the treatment/medication that was proposed to you? What kind of connection is there?" (Ability to integrate information)

8. "Can you tell me the relationship between the immediate effects and the long-term consequences of your condition/problem? (Ability to integrate information)

9. "In general, have you ever received an explanation from the doctor about your health and misinterpreted it? If it happened, did you realize it while continuing to talk to the doctor about your misinterpretation? Or did you realize it later or thanks to others? (Ability to construct meaning - confirmation/disconfirmation of expectations)

10. Assess the patient's ability to make semantic inferences (Ability to make semantic inferences) (**Score in the Appendix).

"Now I will tell you some sentences from which I will ask you to draw conclusions. For example, I will now give you a simple definition of a common disease:

- Osteoporosis is a condition that withdraws calcium from the skeleton and leads to a significant loss of bone mass. Bones are therefore more fragile and prone to fractures. Osteoporosis is difficult to recognize as it is generally symptom-free for many years. It is indeed called the 'silent thief.' Why do you think we call it a silent thief?

- In an advertising campaign to increase donations for research, the following phrase emerged as the winner: 'Life is an apple, cancer its worm, research its pesticide'. What do you think is the message contained in this phrase?

- From the phrase 'everything that is not eaten is good for health', what can we conclude?".

**Information on Appreciating**

1. "In your opinion, what are the advantages/disadvantages of seeking medical treatment (or not seeking treatment) when you have a health issue?" (Ability to assess pros and cons).

2. "If a treatment/medication is proposed to you, are the pros and cons always clear to you? Give me an example" (Ability to assess pros and cons).

3. "Have you understood how the effects of the treatment can impact your daily life?" (Ability to assess the implications of pros and cons).

4. "When you take medication or undergo a treatment, whose advice do you usually seek?" (Ability to weigh the source of information).

5. "In your opinion, if a treatment is suitable for your condition, will it necessarily be effective for you?" (Ability to weigh uncertainty).

6. "When deciding whether to take medication or undergo a treatment/intervention, do you only consider the final outcome, or do you also consider the possibility of failure?" (Ability to weigh uncertainty).

7. "If the doctor tells you something that is not clear, what do you do? Do you ask more specific questions? Do you seek information from other sources (e.g., the Internet, package insert, relatives, friends, newspapers, etc.)?" (Ability to actively seek information).

8. "When deciding to take/not take medication or undergo/not undergo treatment, do you only consider the consequences for yourself, or do you decide based on the impact your choice will have on others (e.g., family, colleagues, etc.)? Can you provide examples?" (Ability to assess the implications of one's choices on others).

9. "When making a choice regarding your health, do you think about the immediate effect, or do you evaluate the long-term effects? Can you provide examples?" (Ability to assess consequences over time).

**Information on Reasoning**

1. "Once you have received all the information about your condition and the possible pros and cons of therapy, based on what do you decide?" (Ability to reason about pros and cons).

2. "If two alternative treatments were proposed to you, each with its own pros and cons, how would you make a decision? Have you ever found yourself in a similar situation?" (Ability for logical reasoning).

- If yes, encourage the patient to share the episode.

- If no, provide an example and invite the patient to reason. “Here's an example I propose: suppose you have hypertension, and you have to decide between two drugs I suggest; the first is drug X, which lowers your blood pressure and reduces the risk of a heart attack but may cause fainting; the second is drug Y, which also lowers your blood pressure and reduces the risk of a stroke but may cause annoying cough. Based on what, would you choose one drug over the other?".

3. "On a scale from 0 to 100, how confident do you feel that you have simultaneously considered all aspects of the choice you made? (Awareness of the logical process leading to the decision).

**Information on Expressing a choice**

1. Investigate during the interview the responses and motivations that the patient reports regarding health-related issues (Volition and Motivation).

2. "When you experience symptoms or realize that something is not right with your health, do you spontaneously go to the doctor? Or do you only go when others, such as your family, push you? Do you undergo all the examinations that are requested of you? Do you adhere to the doctor's prescriptions?" (Volition).

3. "When you have a health problem, do you generally make a personal choice, or do you prefer to delegate to others? If you prefer to delegate, can you explain why?" (Volition).

4. "Typically, are you interested in making decisions about your health, or do you prefer not to worry about it?" (Motivation).

5. "Have you ever found yourself unable to decide between various alternatives that the doctor proposed regarding a drug/medical treatment/surgical intervention? If it has happened, were you eventually able to make a decision? (Ability to manage ambivalence).

****Appendix**

**Semantic Inferences Scores**

4 pts: The subject appropriately identifies the meaning of the inference and can extract its content.

3 pts: The subject interprets the inference adequately but only after self-correction or manages to abstract but provides only a partially relevant explanation.

2 pts: The subject interprets the inference in the literal sense.

1 pt: The subject says something vaguely related to the inference or repeats, paraphrasing the interviewer's questions or statements.

0 pts: The subject does not respond, says something unrelated to the inference, or repeats in a literal manner what the interviewer said.

Assign a score for each of the three semantic inferences and calculate the average to assign the final score (Ability to make semantic inferences).

**The Evaluation of Informed Consent to Treatment (EICT) Scale**

**1. Information on Understanding**

|  | Absent | Uncertain | Sufficient | Good | Excellent |
| --- | --- | --- | --- | --- | --- |
| Ability to summarize and hierarchize information | 0 | 1 | 2 | 3 | 4 |
| Ability to integrate information | 0 | 1 | 2 | 3 | 4 |
| Ability to construct meaning - confirmation/disconfirmation of expectations | 0 | 1 | 2 | 3 | 4 |
| Ability to make semantic inferences | 0 | 1 | 2 | 3 | 4 |
| Overall score | 0 | 1 | 2 | 3 | 4 |

**2. Information on Appreciating**

|  | Absent | Uncertain | Sufficient | Good | Excellent |
| --- | --- | --- | --- | --- | --- |
| Ability to assess pros and cons | 0 | 1 | 2 | 3 | 4 |
| Ability to assess the implications of pros and cons | 0 | 1 | 2 | 3 | 4 |
| Ability to weigh the source of information | 0 | 1 | 2 | 3 | 4 |
| Ability to weigh uncertainty | 0 | 1 | 2 | 3 | 4 |
| Ability to actively seek information | 0 | 1 | 2 | 3 | 4 |
| Ability to assess the implications of one's choices on others | 0 | 1 | 2 | 3 | 4 |
| Ability to assess consequences over time | 0 | 1 | 2 | 3 | 4 |
| Overall score | 0 | 1 | 2 | 3 | 4 |

**3. Information on Reasoning**

|  | Absent | Uncertain | Sufficient | Good | Excellent |
| --- | --- | --- | --- | --- | --- |
| Ability to reason about pros and cons | 0 | 1 | 2 | 3 | 4 |
| Ability for logical reasoning | 0 | 1 | 2 | 3 | 4 |
| Awareness of the logical process leading to the decision | 0 | 1 | 2 | 3 | 4 |
| Overall score | 0 | 1 | 2 | 3 | 4 |

**4. Information on Expressing a choice**

|  | Absent | Uncertain | Sufficient | Good | Excellent |
| --- | --- | --- | --- | --- | --- |
| Volition (Ability to take initiative voluntarily and to express and control behaviors) | 0 | 1 | 2 | 3 | 4 |
| Motivation | 0 | 1 | 2 | 3 | 4 |
| Ability to manage ambivalence | 0 | 1 | 2 | 3 | 4 |
| Overall score | 0 | 1 | 2 | 3 | 4 |

**Total** (sum of the overall scores of the 4 subscales): ___

***Supplementary Tables***

| **Table S1 - Comparison between ‘Sz-A’ and ‘Sz-NA’ groups for demographic, clinical, competence, and neuropsychological characteristics** | | | | |
| --- | --- | --- | --- | --- |
| **Variable** | **‘Sz_A’ mean ± SD** | **‘Sz_NA’ mean ± SD** | **t-test** | **p-value** |
| Age (years) | 42,55 (±7,7) | 41,52 (±10,72) | 0,283 | 0,779 |
| Age of onset (years) | 23,00 (±5,89) | 23,23 (±6,47) | -0,095 | 0,925 |
| Duration of Illness (years) | 18,7 (±7,7) | 18 (±10,2) | 0,193 | 0,849 |
| Education (years) | 12,91 (±2,43) | 12,43 (±2,92) | 0,466 | 0,644 |
| Olanzapine equivalents (mg) | 12,78 (±12,15) | 21,47 (±37,17) | -0,675 | 0,507 |
| **EICT Total score** | 9,98 (±1,57) | 5 (±2,35) | 7,301* | **< 0,001** |
| **Sub. EICT Und.** | 2,34 (±0,37) | 1,17 (±0,74) | 6,077 | **< 0,001** |
| **Sub. EICT Eval.** | 2,45 (±0,48) | 1,29 (±0,71) | 5,609* | **< 0,001** |
| **Sub. EICT Reas.** | 2,73 (±0,47) | 1,30 (±0,78) | 6,587* | **< 0,001** |
| **Sub. EICT Exp.** | 2,45 (±0,69) | 1,23 (±0,57) | 5,457* | **< 0,001** |
| **SICIATRI-R** | 4 (±0) | 1,78 (±1,81) | 5,882* | **< 0,001** |
| **Sub. MacCAT-T Und.** | 4,79 (±0,73) | 3,70(±1,58) | 2,742 | **0,010** |
| **Sub. MacCAT-T Eval.** | 3,73 (±0,47) | 2,26 (±1,39) | 4,554 | **< 0,001** |
| **Sub. MacCAT-T Reas.** | 6,27 (±0,79) | 3,46 (±2,04) | 5,786 | **< 0,001** |
| **Sub. MacCAT-T Exp.** | 1,73 (±0,47) | 0,93 (±0,77) | 3,123 | **0,004** |
| RAVLT-I | 31,64 (±14,12) | 31,91 (±11,75) | -0,062 | 0,951 |
| RAVLT-D | 9,95 (±13,48) | 5,27 (±3,74) | 1,564 | 0,128 |
| Raven-CPM | 29,23 (±4,47) | 26,44 (±5,55) | 1,455 | 0,155 |
| TMT-A (sec) | 67,40 (±21,65) | 87,78 (±43,58) | -1,397 | 0,172 |
| TMT-B (sec) | 177,50 (±129,82) | 192,04 (±86,67) | -0,380 | 0,707 |
| Semantic verbal fluency | 16,91 (±5,56) | 14,22 (±3,68) | 1,460* | 0,166 |
| **Phonemic verbal fluency** | 33,09 (±10,30) | 25,91 (±8,44) | 2,160 | **0,038** |
| Meta-WCST-Cat | 2,64 (±0,92) | 2,35 (±0,98) | 0,816 | 0,420 |
| Meta-WCST-PE | 5,45 (±6,96) | 6,74 (±5,97) | -0,556 | 0,582 |
| Meta-WCST-LC | 77,64 (±28.03) | 75,80 (±22,53) | 0,205 | 0,839 |
| GSS-2 | 14,45 (±7,14) | 13,83 (±8,28) | 0,216 | 0,830 |
| **Verbal judgments** | 45,73 (±5,51) | 40,17 (±10,16) | 2,061* | **0,048** |
| Map Zoo | 8,18 (±4,07) | 4,30 (±6,61) | 1,781 | 0,084 |
| MT Text A | 7,43(±0,98) | 5,93 (±2,60) | 1,950* | 0,066 |
| MT Text C | 6 (±2,28) | 4,95 (±2,54) | 1,146 | 0,261 |
| MT Text E | 3,73 (±1,62) | 4,77 (±2) | -1,503 | 0,143 |
| MMSE | 28,55 (±1,69) | 28,13 (±1,18) | 0,831 | 0,412 |
| DEX | 24,27 (±12,22) | 30,09 (±20,5) | -0,866 | 0,393 |
| DSES | 66,53 (±16,8) | 68,18 (±22,52) | -0,216 | 0,831 |
| PANSS Positive | 14,36 (±5,08) | 15,00 (±5,29) | -0,332 | 0,742 |
| PANSS Negative | 22,82 (±8,89) | 21 (±4,66) | 0,788 | 0,436 |
| PANSS General | 30,73 (±10,94) | 36,57 (±8,35) | -1,724 | 0,094 |
| PANSS Total | 67,91(±20,83) | 72,57 (±15,37) | -0,116 | 0,153 |
| PANSS Disorganized/Concrete Factor^*^ | 6,18 (±3,12) | 7,57 (±3,5) | -1,114 | 0,274 |
| PANSS Excited Factor^*^ | 5,45 (±4,08) | 6,83 (±2,35) | -1,247 | 0,221 |
| PANSS Depressed Factor^*^ | 5,09 (±2,3) | 6,39 (±2,44) | -1,478 | 0,149 |
| IS | 11,5 (±4,76) | 14,3 (±6,11) | -0,957 | 0,355 |
| AIMS | 0,30 (±0,48) | 0,4 (±0.88) | -0,332 | 0,742 |
| BARS | 0,8 (±1,03) | 0,25 (±) | 1,611* | 0,136 |
| SAS | 4,44 (±3,36) | 3,30 (±2,6) | 1,002 | 0,325 |
| - * Levene’s test p-value < 0,05  - Bold values denote statistical significance at the p < 0.05 level | | | | |

| **Table S2 - Comparison between ‘Sz-Male’ and ‘Sz-Female’ groups for demographic, clinical, competence, and neuropsychological characteristics** | | | | |
| --- | --- | --- | --- | --- |
| **Variable** | **Sz-Male mean ± SD** | **Sz Female mean ± SD** | **t-test** | **p-value** |
| Age (years) | 41,76 ± (10,34) | 42,11 ± (8,35) | -0,091 | 0,928 |
| Age of onset (years) | 22,38 ± (6,05) | 25,50 ± (6,41) | -1,246 | 0,222 |
| Duration of Illness (years) | 19,00 ± (9,73) | 15,88 ± (8,35) | 0,812 | 0,423 |
| Education (years) | 12,56 ± (2,77) | 12,67 ± (2,82) | -0,099 | 0,922 |
| Olanzapine equivalents (mg) | 21,75 ± (35,87) | 11,13 ± (12,20) | 0,807 | 0,428 |
| EICT Total score | 6,96 ± (3,33) | 5,64 ± (2,56) | 1,081 | 0,288 |
| Sub. EICT Und. | 1,66 ± (0,87) | 1,25 ± (0,73) | 1,255 | 0,219 |
| Sub. EICT Eval. | 1,74 ± (0,84) | 1,46 ± (0,86) | 0,851 | 0,401 |
| Sub. EICT Reas. | 1,87 ± (1,02) | 1,44 ± (0,72) | 1,168 | 0,251 |
| Sub. EICT Exp. | 1,68 ± (0,93) | 1,48 ± (0,50) | 0,606 | 0,549 |
| SICIATRI-R | 2,36 ± (1,84) | 2,89 ± (1,76) | -0,745 | 0,462 |
| Sub. MacCAT-T Und. | 4,09 ± (1,48) | 3,96 ± (1,41) | 0,232 | 0,818 |
| Sub. MacCAT-T Eval. | 2,72 ± (1,48) | 2,78 ± (0,97) | -0,108 | 0,915 |
| Sub. MacCAT-T Reas. | 1,64 ± (0,56) | 1,67 ± (0,50) | 0,764 | 0,451 |
| Sub. MacCAT-T Exp. | 4,54 ± (2,18) | 3,88 ± (2,20) | -0,124 | 0,902 |
| **RAVLT-I** | 34,52 ± (12,96) | 44 ± (11) | -2,066 | **0,047** |
| RAVLT-D | 8,16 ± (10,35) | 9,56 ± (3,20) | -0,430 | 0,670 |
| Raven-CPM | 30,48 ± (4,42) | 27,56 ± (6,67) | 1,480 | 0,149 |
| TMT-A (sec) | 80,50 ± (45,02) | 74,56 ± (24,43) | 0,373 | 0,711 |
| TMT-B (sec) | 201,13 ± (110,16) | 151,67 ± (53,95) | 1,061 | 0,297 |
| Semantic verbal fluency | 15,16 ± (4,48) | 14,89 ± (4,70) | 0,154 | 0,879 |
| Phonemic verbal fluency | 27,40 ± (10,02) | 30,56 ± (8,17) | -0,846 | 0,404 |
| Meta-WCST-Cat | 2,40 ± (1) | 2,56 ± (0,88) | -0,412 | 0,683 |
| Meta-WCST-PE | 6,04 ± (6,03) | 7,11 ± (7,07) | 0,192 | 0,849 |
| Meta-WCST-LC | 2,56 ± (0,88) | 63,0 ± (26,1) | 1,867 | 0,086 |
| GSS-2 | 14,24 ± (8,13) | 13,44 ± (7,31) | 0,258 | 0,798 |
| Verbal judgments | 42,40 ± (8,60) | 40,78 ± (11,24) | 0,447 | 0,658 |
| Map Zoo | 5,96 ± (6,14) | 4,44 ± (6,3) | 0,630 | 0,533 |
| MT Text A | 6,17 ± (2,38) | 7,50 ± (1,73) | -1,050 | 0,306 |
| MT Text C | 5,24 ± (2,57) | 5,57 ± (2,22) | -0,309 | 0,759 |
| MT Text E | 4,52 ± (2,00) | 4,13 ± (1,72) | 0,500 | 0,620 |
| MMSE | 28,40 ± (1,38) | 27,89 ± (1,26) | 0,969 | 0,340 |
| DEX | 27,12 ± (19,47) | 31,22 ± (14,89) | -0,572 | 0,571 |
| DSES | 29,68 ± (9,74) | 30,00 ± (7,34) | -0,102 | 0,920 |
| PANSS Positive | 15,12 ± (5,52) | 13,89 ± (4,10) | 0,700 | 0,492 |
| PANSS Negative | 21,60 ± (6,82) | 21,56 ± (4,66) | 0,021 | 0,983 |
| PANSS General | 33,88 ± (10,24) | 36,89 ± (7,14) | -0,958 | 0,349 |
| PANSS Total | 70,60 ± (18,88) | 72,33 ± (11,74) | -0,318 | 0,753 |
| PANSS Disorganized/Concrete Factor^*^ | 6,72 ± (3,57) | 8,22 ± (2,72) | -1,142 | 0,262 |
| PANSS Excited Factor^*^ | 6,56 ± (3,28) | 5,89 ± (2,26) | 0,565 | 0,576 |
| PANSS Depressed Factor^*^ | 5,72 ± (2,40) | 6,67 ± (2,55) | -0,997 | 0,326 |
| IS | 12,85 ± (6,14) | 15,00 ± (2,64) | -0,582 | 0,570 |
| AIMS | 0,30 ± (0,63) | 0,57 ± (1,13) | -0,804 | 0,428 |
| BARS | 0,48 ± (0,79) | 0,29 ± (0,48) | 0,606 | 0,549 |
| SAS | 3,95 ± (2,95) | 2,71 ± (2,43) | 1,005 | 0,324 |
| - * Levene’s test p-value < 0.05  - Bold values denote statistical significance at the p < 0,05 level | | | | |

| **Table S3 - Item Analysis** | | | | | |
| --- | --- | --- | --- | --- | --- |
| **Variable** | **EICT Total score** | **Sub. EICT Und.** | **Sub. EICT Eval.** | **Sub. EICT Reas.** | **Sub. EICT Exp.** |
| Hierarchize information | 0,964^**^ | 0,971^**^ | **/** | **/** | **/** |
| Integrate information | 0,975^**^ | 0,978^**^ | **/** | **/** | **/** |
| Meaning construction | 0,971^**^ | 0,980^**^ | **/** | **/** | **/** |
| Semantic inferences | 0,857^**^ | 0,901^**^ | **/** | **/** | **/** |
| Evaluate Pros and Cons | 0,938^**^ | **/** | 0,961^**^ | **/** | **/** |
| Evaluate implications for and against | 0,960^**^ | **/** | 0,969^**^ | **/** | **/** |
| Active information research | 0,909^**^ | **/** | 0,921^**^ | **/** | **/** |
| Weighting information source | 0,903^**^ | **/** | 0,918^**^ | **/** | **/** |
| Weighing uncertainty | 0,942^**^ | **/** | 0,962^**^ | **/** | **/** |
| Evaluate choices implication | 0,925^**^ | **/** | 0,950^**^ | **/** | **/** |
| Evaluate choices consequences | 0,940^**^ | **/** | 0,943^**^ | **/** | **/** |
| Reasoning about pros and cons | 0,964^**^ | **/** | **/** | 0,972^**^ | **/** |
| Logic reasoning | 0,927^**^ | **/** | **/** | 0,966^**^ | **/** |
| Awareness of choice process | 0,932^**^ | **/** | **/** | 0,979^**^ | **/** |
| Volition | 0,948^**^ | **/** | **/** | / | 0,976^**^ |
| Motivation | 0,920^**^ | **/** | **/** | / | 0,965^**^ |
| Ambivalence management | 0,951^**^ | **/** | **/** | / | 0,972^**^ |
| ** Significant at p <0,001 | | | | | |
